# Stinging Nettle (*Urtica dioica* L.) as a Functional Component in Durum Wheat Pasta Production: Impact on Chemical Composition, In Vitro Glycemic Index, and Quality Properties

**DOI:** 10.3390/molecules26226909

**Published:** 2021-11-16

**Authors:** Ada Krawęcka, Aldona Sobota, Urszula Pankiewicz, Ewelina Zielińska, Piotr Zarzycki

**Affiliations:** 1 Department of Plant Food Technology and Gastronomy, University of Life Sciences in Lublin, Skromna 8 Street, 20-704 Lublin, Poland; ada.krawecka@gmail.com (A.K.); piotr.zarzycki@up.lublin.pl (P.Z.); 2 Department of Analysis and Evaluation of Food Quality, University of Life Sciences in Lublin, Skromna 8 Street, 20-704 Lublin, Poland; urszula.pankiewicz@up.lublin.pl (U.P.); ewelina.zielinska@up.lublin.pl (E.Z.)

**Keywords:** enriched pasta, functional food, glycemic index, phytochemicals, pigments, minerals, dietary fiber

## Abstract

Stinging nettle (*Urtica dioica* L.) is a good source of biologically active compounds with proven beneficial health effects. This study aimed to investigate the effect of nettle herb supplementation on chemical composition, including the content of selected minerals and pigments, the in vitro glycemic response, and the cooking and sensory quality of extruded pasta. Tagliatelle-shaped pasta was produced under semi-technical scale by partial replacement of durum wheat semolina with 0, 1, 2, 3, 4, and 5% of lyophilized nettle. The partial substitution with freeze-dried nettle caused a statistically significant (*p* ≤ 0.05) increase in the content of minerals, especially calcium, iron, potassium, and magnesium in the products. The calcium content in the pasta fortified with 5%-addition of stinging nettle was 175.9 mg 100 g^−1^ and this concentration was 5.8 times higher than in the control sample. At the same time, high content of chlorophylls and carotenoids (237.58 µg g^−1^ and 13.35 µg g^−1^, respectively) was noticed. Enriching pasta with a 0–5% addition of stinging nettle resulted in a statistically significant (*p* ≤ 0.05) increase in the content of the total dietary fiber (TDF) (from 5.1 g 100 g^−1^ to 8.82 g 100 g^−1^) and the insoluble dietary fiber (IDF) (from 2.29 g 100 g^−1^ to 5.63 g 100 g^−1^). The lowest hydrolysis index of starch (HI = 17.49%) and the lowest glycemic index (GI = 49.31%) were noted for the pasta enriched with 3% nettle.

## 1. Introduction

Stinging nettle (*Urtica dioica* L.) is a representative of the nettle family (*Urticaceae*). It is an annual plant and it grows mainly wild. Stinging nettle is widely popular in Europe, Asia, North America, and North Africa. Its healing properties were widely known in ancient times and were described by the father of medicine, Hippocrates [1,2]. Nettle is usually associated with its diuretic properties and the treatment of urinary tract infections. In addition, antioxidant, anti-inflammatory, hypoglycemic, and hypocholesterolemic properties are attributed to it. This activity is the result of biologically active compounds present in nettle such as phenolic acids (including protocatechuic, quinic, coumaric, coffee, ferulic), tannins, pigments, unsaturated fatty acids, sterols, and phytoestrogens [1,3,4,5].

Pasta belongs to popular and frequently eaten cereal products. It occurs in many different forms and shapes and its high durability, easy preparation for consumption, relatively low price, and nutritional quality determine its attractiveness among modern consumers. Extruded pasta produced with durum semolina is characterized by a low glycemic index. The unique structure of pasta, in which starch is tightly surrounded by a protein matrix, hinders the activity of amylolytic enzymes and thus slows down the dynamics of glucose release and reduces postprandial glycemia [6]. In addition, pasta is a food product that can be easily fortified with plant raw materials rich in phytochemicals with hypoglycemic, antioxidant, or hypocholesterolemic effects. Stinging nettle is one such raw material. Thus far, it has been successfully used as a functional ingredient of bread [7,8] or as a component of a herbal mixture added to wheat bread baked without salt [9]. It has also been used to coagulate milk proteins in the cheese-making process [10] and concentrated and lyophilized stinging nettle extract was used to manufacture chocolates [11]. In order to enhance the activity of bioactive compounds contained in nettle, attempts have been made to nanocapsulate its extracts and the research findings indicate the possibility of using the obtained nanocomposites in various food matrices [12,13]. Nettle is a raw material not only of a high health-promoting potential. Rich in chlorophyll, it can be used beside vegetable powders and concentrates as a natural coloring component [14,15].

The effect of a large addition (5–20%) of nettle on the chemical composition and sensory acceptance of wheat noodles was investigated by Alemayehu et al. [16]. The authors produced pasta on a small laboratory scale using lamination technology. In the research, *Urtica simensis* growing in Africa was used. There are no studies on the impact of the lower (1–5%) addition of the most popular nettle species, which are growing in Eurasia (*Urtica dioica* L.) on the nutritional and quality properties of extruded durum wheat pasta. The purpose of this study was to determine the possibility of using a low concentration of stinging nettle to obtain functional wheat durum pasta with improved health-promoting value and with a high cooking and sensory quality at the same time.

## 2. Results and Discussion

### 2.1. Pasta Processing

The addition of stinging nettle affects the extrusion process of the pasta. The increasing content of stinging nettle in the pasta enriched with 1–5% resulted in higher pressure values, compared to the control sample (Table 1). At the same time, the addition of nettle increased extruder output. The trend noted may be due to changes in the rheology of the pasta dough. The introduction of high-fiber components that are characterized by high water absorption and compete for water with durum semolina, hinders the proper hydration of pasta dough ingredients and affects its lower plasticity [17]. As a consequence, a simultaneous increase in the extrusion pressure and extruder output was observed (Table 1).

### 2.2. Basic Chemical Analysis

The chemical composition of the pasta samples is reported in Table 2. One of the most important chemical components determining the quality characteristics of pasta is gluten protein. After the ingredients of the pasta dough are hydrated, it forms a characteristic gluten network that tightly surrounds the swollen starch granules [6]. Despite the significantly higher amount of protein in the stinging nettle raw material than in the semolina durum, the 1–5% addition of the stinging nettle did not affect the protein content in the enriched samples compared to the control sample. The levels of fortifications used turned out to be insufficient for a significant increase in this parameter. Slightly different results were obtained by Alemayehu et al. [16]. Those researchers enriching wheat noodles with a 5–20% addition of stinging nettle noted a statistically higher (*p* < 0.05) protein content compared to the control sample. The free fat content also did not change significantly as a function of an increasing nettle addition. However, attention should be paid to a significantly lower content of free fat in all pasta samples compared to the raw materials. The observed trend is consistent with the results presented in the literature. During the production of pasta at the pressing stage, but above all at the drying stage, the fat is complexed by starch and protein. The degree of fat complexation can be as high as 25% [18]. Based on presented results of the free fat content (Table 2), it was estimated that 81–86% of free fat complexed during the production of the pasta samples. A similar trend was noted by Alemayehu et al. [13], who fortified noodles with 5–20% of dried nettle. The results of this study indicate that the increasing share of nettle promotes lipid complexation. This could affect the digestibility of starch and the glycemic index (GI) of pasta. The amylose–lipid complexes, defined as resistant starch (RS5), are not digested in the gastrointestinal tract and may affect the lower glycemic index of products [19]. Nettle, compared to semolina, is rich in minerals [2]. Along with the increase in stinging nettle addition, the ash content increased significantly (*p* ≤ 0.05). The pasta sample fortified with 5% stinging nettle (N5) characterized by 2 times higher concentration of ash than the control sample (CON). Similarly, Đurović et al. [7], when using nettle leaves and/or nettle extract for bread making, found that the addition of 5% of dried leaves resulted in a significant increase in the ash content (1.83% vs. 1.06% for the control) and only a slight increase in the protein content (11.65% for the sample with 5% share of nettle leaves vs. 11.14% for the control). The literature review shows that stinging nettle is a good source of dietary fiber [2,8]. The total dietary fiber (TDF) content in stinging nettle is more than 10 times higher than in semolina durum. The addition of stinging nettle at the levels proposed in our study caused a significant (*p* ≤ 0.05) increase in the TDF content and also in the insoluble dietary fiber (IDF) content. There were no significant differences in the soluble fraction (SDF) content. In the studies by Alemayehu et al. [16], the content of crude fiber in the pasta samples increased significantly (*p* < 0.05) from 1.25 to 2.65%, along with an increase in the share of nettle from 5 to 20%. It should be noted that the reported values were much lower than the TDF content reported in our study for samples with 1–5% addition of stinging nettle. The estimated digestible carbohydrates content in the enriched pasta decreased compared to control sample (CON). A decrease in the content of digestible carbohydrates was observed by other authors in the case of wheat bread enriched with nettle leaves [8]. Products with a lower content of digestible carbohydrates should induce a lower glycemic response and thus positively affect carbohydrate metabolism and diabetes prevention [6].

### 2.3. Determination of Calcium, Iron, Potassium Magnesium, and Phosphorus Concentration

Stinging nettle contains large amounts of calcium, iron, potassium, magnesium, as well as silicon and phosphorus [20,21]. The chemical composition of the nettle herb is determined by the harvest period. It was found that the plant harvested in April and May contained a higher content of soluble solids and some minerals—phosphorus, potassium, iron, zinc—and it has the highest antioxidant activity [22]. The content of selected minerals for the raw materials and pasta samples is reported in Table 3. Along with the increase in the amount of stinging nettle, a significant (*p* ≤ 0.05) increase in the content of calcium, iron, potassium, and magnesium was observed. The increase in the calcium content was 5.8 times higher in the sample enriched with 5% of stinging nettle (N5) than in the control sample (CON). As regards iron, the highest value was observed also for the N5 sample. Consumption of 100 g pasta enriched with 5% nettle covers approximately 20% recommended daily intake (RDI) of calcium and iron. For the potassium and magnesium content, statistically significant (*p* ≤ 0.05) differences compared to the control sample began to appear at the 2% and 3% enrichment levels (N2 and N3 samples, respectively). The significant increase in the concentration of the minerals was highly correlated (r > 0.8, *p* < 0.05) with the ash and total dietary fiber content (TDF) in the enriched pasta samples (Table 2). A few non-significant differences were noted in the content of silicon and phosphorus. The results of our research are consistent with the results obtained by Alemayehu et al. [16]. Those researchers noted a 5 times higher value of calcium for the pasta sample with a 5% addition of stinging nettle compared to the control sample (wheat flour pasta). Additionally, the content of iron and zinc increased in the enriched samples. The high content of minerals in food enriched with nettle means that it can be used in diets for those at risk of osteoporosis or cardiovascular diseases. The fact that nettle contains significant amounts of vitamin C means that the iron contained in it will be characterized by increased bioavailability [23].

### 2.4. Determination of the Chlorophyll and Carotenoid Content

Chlorophyll is the main photosynthetic pigment in nettle. Apart from the fact that it determines the intense green color of nettle, it also has a bioactive effect. It is believed to have detoxifying properties and support cleansing of the digestive tract. In addition, it prevents flatulence and bad breath [23]. The literature data indicate that the content of chlorophyll and carotenoids in food products is positively and significantly correlated with antiradical activity. The ratio of chlorophyll *a* to chlorophyll *b* is usually 3:1 [15]. In the tested semolina durum and stinging nettle, this ratio was different and amounted to 6.5:1 and 1.4:1, respectively. There are also several carotenoids with antioxidant properties in nettle, including β-carotene, xanthophyll, lutein, and lycopene [1]. The pigment content is determined by the climate and the environment. Larger amounts of pigment are found in plants growing in shaded conditions [2]. Pigments are sensitive to light, temperature, enzyme action, and changes in pH values [15]. The results of our research (Table 4) indicate a significant (*p* ≤ 0.05) increase in the content of pigments in the tested pasta samples compared to the control sample. Samples enriched with 5% stinging nettle were characterized by high chlorophyll and carotenoid content (237.58 µg g^−1^ and 13.35 µg g^−1^, respectively), which also indicates their higher antioxidant potential. Statistically significant (*p* < 0.05) increases in the chlorophyll and carotenoid content were noted by other researchers after the inclusion of spirulina (with the addition of 1–3%), also used as a natural pigment for gluten-free pasta based on rice flour [24].

### 2.5. Cooking Quality

Previous studies indicate that additions of raw materials with a high content of dietary fiber may extend the cooking time and the cooking loss [25,26]. This study revealed a significant (*p* ≤ 0.05) increase in the cooking time (CT) for the enriched samples (Table 5). The research confirmed high positive correlation (r = 0.97, *p* < 0.05) between the cooking time of pasta and total dietary fiber content (TDF). Man et al. [8] observed that the addition of dried nettle to wheat bread had a limiting effect on the quality parameter, i.e., the volume of bread, and related it to the weakening of the gluten network in the blend and the interactions among fiber components, water and gluten. Nevertheless, the enriched samples showed at least as good sensory acceptance as the control bread sample, in terms of bread volume. Đurović et al. [7] found that the addition of nettle leaves in bread reduced its quality while the nettle extract had a positive effect on the quality parameters. Along with the increase in stinging nettle, the cooking loss values of the cooked pasta samples increased significantly (*p* ≤ 0.05) (Table 5). This may be due to disintegration and weakening of the gluten network as a result of incorporation of the high-fiber component. The changes in the gluten matrix may favor the rinsing of starch during the hydrothermal treatment of pasta. Our research confirmed the high positive correlation (r = 0.96, *p* < 0.05) between the total dietary fiber content and the loss of dry matter. Pasta is considered unacceptable if the loss of dry matter is ≥8%. It should be remembered that the size of this parameter is influenced, among others, by the specific surface of the pasta (shape and size of pasta) as well as specificity of the raw material (particle size, degree of starch damage) and their chemical composition. Yu et al. [27] indicated that larger particles of green tea powder introduced into noodles had the effect of increasing the loss of dry matter while cooking pasta. Moreover, the shape and form of pasta also influence the stability of the product during the cooking process. In the above study, the shape of the tagliatelle-shaped ribbon used could cause a greater degree of leaching of soluble substances as a result of the increase in the area of contact of pasta with water. Extending cooking time and increasing dry matter losses were also observed by Teterycz et al. [28] by introducing hemp flour in durum wheat pasta. In contrast, Sobota et al. [14] noted increased losses of dry matter as a result of adding vegetable powders and concentrates to pasta. The addition of stinging nettle affects only slight increase in the weight of pasta under cooking (Table 5). 

### 2.6. Determination of Glycemic Index (GI) In Vitro

**Figure 1:**
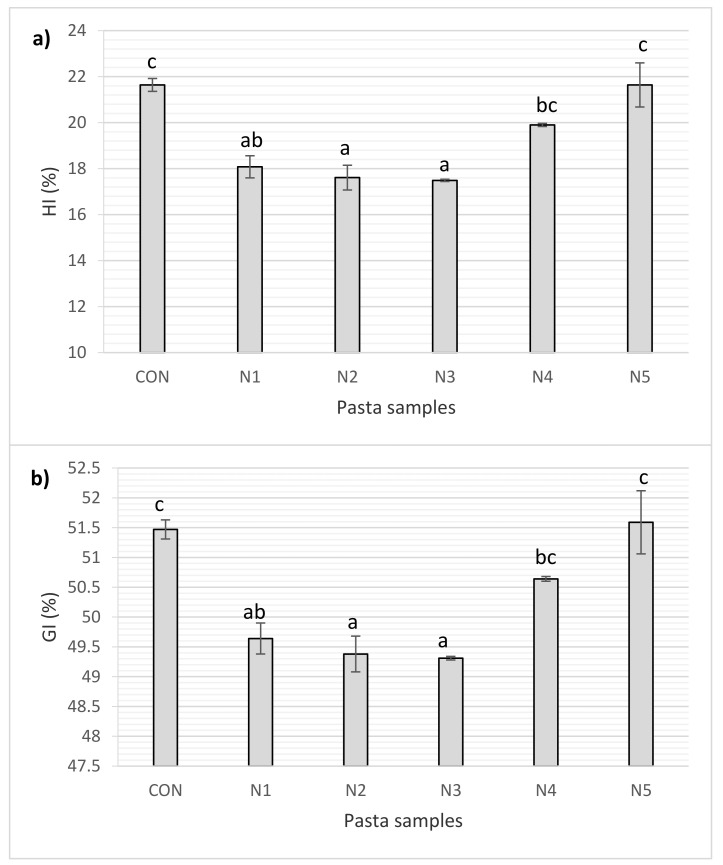
The in vitro starch hydrolysis index (**a**) and glycemic index values (**b**) of pasta. Explanation: HI—hydrolysis index of starch; GI—glycemic index values; CON—control sample; N—pasta with stinging nettle. Data are presented as mean (*n* = 2) ± standard deviation. Data values of each parameter with different superscript letters are significantly different (Tukey’s test, *p* ≤ 0.05).

An in vitro starch hydrolysis method was used to estimate the metabolic glycemic response to food products. The in vitro hydrolysis index of starch (HI) and glycemic index values (GI) of pasta are shown in Figure 1. GI is defined as low if it is less than 55% [29]. It should be stressed that the predicted glycemic index of the control pasta sample (CON), produced in 100% from durum semolina, was low and amounted to 51%. Many researchers emphasize that pasta is a carbohydrate product with a more beneficial effect on glycemia than bread. The meta-analysis study by Huang et al. [30] shows that pasta induces a statistically significant (*p* ≤ 0.05) milder glycemic response than wheat bread and potatoes. In our research, it was noticed that the lowest total starch hydrolysis (HI) was observed in pasta enriched with nettle at the level of 2% and 3%. The addition of stinging nettle affected the GI values of pasta. The lowest GI values (49.31, 49.38, and 49.64%) were achieved for pasta enriched with 3, 2, and 1% addition of stinging nettle, respectively. With the substitutions of 4 and 5%, the GI values did not differ from the control sample. Increasing the nettle content weakens the gluten network, which may favor greater availability of starch for amylolytic enzymes and translate into higher digestion dynamics. The weakening of the gluten network is confirmed by the results of research on the cooking quality of pasta (Table 5). Nettle is characterized by a low glycemic index [2]. The content of dietary fiber, tannins, and total polyphenols may induce functional effects in terms of digestion and absorption of carbohydrates. Phenolic compounds can inhibit amylolytic enzymes and delay glucose absorption, contributing to lowering postprandial glycemia [31,32]. Black mulberry, whose extract was used as a functional additive in pasta, is equally rich in phenolic compounds, especially anthocyanins, flavonols, and chlorogenic acid [33]. Black mulberry extract inhibited in vitro starch hydrolysis and lowered the predicted GI of pasta while increasing its antioxidant activity. Pistachio green husk extract was also successfully used as a functional additive to pasta [34]. Among the polyphenols contained in pistachio shells, gallic acid, cinnamic acid, and catechins were isolated. The in vitro digestibility of starch and the predicted GI of the fortified pasta were significantly (*p* < 0.05) lower than the control sample pasta. For the highest extract addition used (1.5%), the GI value was 36.17%. The GI values obtained for pasta with 0.5% extract (48.72%) were similar to those in our research for pasta enriched at the level of 1–3% (49.31–49.64%) (Figure 1).

### 2.7. Sensory Quality

**Figure 2:**
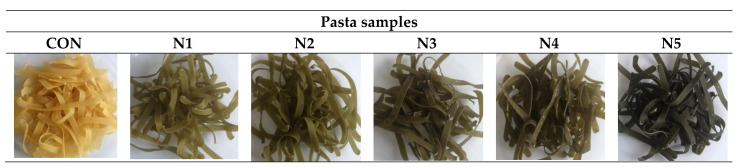
Pasta with 0–5% addition of stinging nettle. Explanation: CON—control sample; N—pasta with stinging nettle.

The appearance, taste, and texture of the product, including its adhesiveness, have a great influence on the acceptance of the product by consumers. The pasta samples with 0–5% addition of nettle are presented in Figure 2. The results of the sensory assessment confirmed that even the 5% addition of stinging nettle did not deteriorate the sensory quality of the uncooked and cooked products (Table 6). All the enriched uncooked pasta samples were approved by the panelists. Slightly significant differences were found as regards appearance, color, and odor between the uncooked products enriched with stinging nettle (sample N1 and N2) and the control sample (CON).

Although in the case of cooked pasta, there were no significant differences (*p* ≤ 0.05) in the appearance, hardness, adhesiveness, and springiness between stinging nettle-supplemented samples and the control samples (CON). The tastes of the pasta with 3% and 5% addition of stinging nettle, respectively, were noted significantly (*p* ≤ 0.05) lower than the control sample. Other researchers [16] found that the optimal addition of dried nettle leaves to wheat noodles should not be greater than 10%. With the increase in the supplementation level, a significant deterioration in the sensory quality was observed. The acceptability of color, smell, texture, and taste decreased significantly (*p* < 0.05), which negatively affected the overall acceptability of pasta enriched with 15% and 20% nettle. Sobota et al. [14], by adding vegetable powders and concentrates to pasta, found that the flavor of fortified pasta (beetroot, carrot, and kale) scored slightly worse than the durum semolina control pasta. The most intense changes in taste were recorded for pasta with the addition of kale. However, as in the authors’ research, the differences were not statistically significant in every product. In our research, the addition of nettle at the level of 4 and 5% had a positive effect on the color assessment in the case of cooked pasta. In the studies by Belščak-Cvitanović et al. [11], the addition of nettle extracts (in a freeze-dried or concentrated form) to chocolate improved sensory parameters. The use of freeze-dried extract increased in the content of polyphenols, and polyphenols include many compounds of the nature of dyes or flavorings, which may translate into the observed sensory characteristics.

## 3. Material and Methods

### 3.1. Characteristics of Raw Materials

Durum semolina (Julia Malom, Kunszállás, Hungary) and lyophilized nettle herb were used for production of pasta. The lyophilizate was obtained from the above-ground part of the nettle collected during the budding phase in the Lublin region, Poland in April and May. The raw material was pre-ground, frozen at −20 °C, freeze-dried with a freeze-drier, then ground and stored in plastic bags at −20 °C. Pasta samples were produced with 100% of semolina durum (CON) and by replacing durum wheat semolina by 1, 2, 3, 4, and 5% of lyophilized nettle (samples N1, N2, N3, N4, N5, respectively). The detailed model of the experiment is presented in Table 1.

### 3.2. Pasta Preparation

An MAC-30S Lab pasta extruder (ItalPast, Parma, Italy) and EAC30-LAB pasta dryer (ItalPast, Parma, Italy) were used to produce tagliatelle-shaped pasta under semi-technical conditions. Constant addition of water was applied amounting to 333 mL·kg^−1^. First, ingredients were mixed for 15 min under atmospheric pressure and then dough was mixed under vacuum and extruded under the pressure given in Table 1. The rotational speed of the screw of the pasta extruder was 48 rpm. The pasta samples were dried at a controlled temperature and humidity. The detailed conditions of this process were described by Sobota et al. [14]. 

### 3.3. Basic Chemical Analysis

The analysis of the chemical composition was performed using AACC and AOAC methods [35,36]. 

Moisture content was determined using the Air-oven method (Method AACC 44-15A). The samples (3 g) were placed in a laboratory dryer and dried at 103 °C ± 1 °C to a constant weight. After cooling in an exsiccator, the samples were weighed, and the moisture contents were calculated. 

Ash content was determined using AACC method 08–01. The samples were measured into ash dishes in amounts of 3 g and then placed in a muffle furnace at 550 °C. They were incinerated until light gray ash or constant weight was obtained (7 h). After cooling, the samples were weighed, and the ash contents were calculated. 

Total protein content was measured using the Kjeldahl method (Method AACC 46-08). The protein content was calculated from the total nitrogen content where a converted factor of 6.25 was used. 

Free fat content was determined by continuous extraction. The SoxtecTM8000 on application AN 310 (FOSS, Höganäs, Sweden) and hexane as a solvent were used. 

Total dietary fiber *(TDF*) content, including insoluble dietary fiber (IDF) and soluble dietary fiber (SDF) were determined according to the enzymatic methods (AACC 32-05, AACC 32-21, AOAC 991.43, and AOAC 985.29). Dried samples, each of 1 g, were subjected to sequential enzymatic digestion by heat-stable α-amylase, protease, and amyloglucosidase. Megazyme enzymes and analytical procedures were used (Megazyme International Ireland Ltd., Wicklow, Ireland). 

Digestible carbohydrate content was determined by calculation of the difference: 100 − (weight in grams [protein + fat + TDF + ash] in 100 g of dry matter of pasta or raw material).

### 3.4. Determination of Calcium, Iron, Potassium, Magnesium, and Phosphorus Concentration

#### 3.4.1. Calcium, Iron, Potassium, Magnesium, and Silicone Concentration

The pasta samples were mineralized in an MARS microwave oven (CEM Corporation, USA). The samples were prepared as follows: about 0.5 g of pasta was transferred into a tube and 4 mL of HNO_3_ was added. Then, the samples were mineralized at 200 °C for 20 min. The obtained solutions were cooled down, transferred to 25 mL measuring flasks, and topped up with deionized water. The concentration of calcium, iron, potassium, and magnesium ions in the pasta was determined using a flame atomic absorption spectrophotometer (FAAS, Solaar939, Unicam, Cambridge, UK). The concentration of silicone ions in the pasta was determined using an ICP-MS.

#### 3.4.2. Phosphorus Concentration

The analytical weight of the sample was incinerated with calcium carbonate and heated with hydrochloric acid and nitric acid (V). Part of the acidic solution was mixed with a molybdenum reagent and the absorbance of the resulting yellow solution was measured at 430 nm with a Shimadzu UV-1800 spectrophotometer. The methodology used during the indications is included in the standard ISO 6491:2000 [37].

### 3.5. Determination of the Chlorophyll and Carotenoid Content

The analysis was performed by the spectrophotometric method using a spectrophotometer Thermo Spectronic (Helios Epsilon, Pittsford, NY, USA). The content of carotenoids (car) and chlorophylls (chl *a* and *b*) was determined according to the method described previously by Złotek et al. [38]. Pigments were extracted from the raw materials and ground pasta samples (0.2 g) overnight with 8 mL 80% (*v*/*v*) acetone at 4 °C. Then, the extracts were centrifuged by 13,000× *g* for 5 min. The absorbance (A) for supernatant was measured in triplicate at 663, 645, and 470 nm. The chlorophyll a, chlorophyll b, and carotenoid contents were calculated from the equations:

chl *a* = 12.72·*A*_663_ − 2.59·*A*_645_
chl *b* = 22.88·*A*_645_ − 4.67·*A*_663_
car = (1000·*A*_470_ − 3.27·chl *a* − 104·chl *b*)/229
and expressed in μg·g^−1^ d.m.

### 3.6. Cooking Quality of Pasta Samples

Optimal cooking time (*OCT*) was measured according to AACC 16-50 Method [35]. A 50 g measure of pasta was boiled in 500 mL of distilled water. Every 30 s, the pasta was removed and squeezed between two glass plates until the mealy core disappeared. The time needed for this process was assumed as optimal cooking time (OCT). 

Weight increase index (WII) was calculated by dividing the weight of the pasta sample after cooking by the weight of an uncooked pasta sample (50 g).

Volume increase index (VII) was tested by dipping a 50 g sample of an uncooked product in a measuring cylinder filled with 400 mL of vegetable oil. The volume increase was equal to the volume of the tested pasta sample. A sample of pasta (50 g) was then cooked and the volume of the cooked product was determined similarly. VII was calculated by dividing the volume of the cooked pasta by the volume of the uncooked product. 

Cooking loss (CL), expressed in g 100 g^−1^ of dry matter, was determined by testing the dry matter content in water after cooking a 50 g pasta sample. The dry matter content in water was determined according to the AACC 44-15A method [35].

### 3.7. Determination of In Vitro Glycemic Index (GI)

The in vitro glycemic index (GI) of pasta was determined by evaluating the in vitro starch digestibility according to the method of Monro et al. [39] with slight modifications. The whole digestion process was carried out at 37 °C with stirring (130 rpm) in darkness. Briefly, 30 mL of water and 0.8 mL of 1 M HCl were added to 1 g of the sample to reach the pH of 2.5. Subsequently, gastric digestion was initiated by adding 1 mL of 10% pepsin solution in 0.05 M HCl. After 30 min, the small intestine phase was started. In this regard, 2 mL of 1 M NaHCO_3_ and 5 mL of 0.1 M phosphate buffer (pH 6) were added, and then 4.6 mg amyloglucosidase and 5 mL of 2.5% pancreatin in 0.1 M phosphate buffer (pH 6). The volume of hydrolysates was adjusted of distilled water to 55 mL. According to the Reis and Abu-Ghannam method [40] after 20, 30, 60, 90, 120, and 180 min from the start of amylolysis 1.0 mL of digesta aliquots were transferred to 4 mL absolute ethanol so as to inactivate the enzymes. In order to determine the glucose content in the samples (mg glucose/g sample), the GOPOD method was used. The glucose content was plotted as a function of time, and the areas under the hydrolysis curves (AUC) were calculated. The hydrolysis index (HI) was calculated as the ratio between the AUC of the sample and the AUC for the reference food (white bread). The glycemic index was predicted according to the formula defined by Goñi, Garcia-Alonso, and Saura-Calixto [41]:

GI (%) = 39.71 + 0.549 × HI

### 3.8. Sensory Quality

The analysis was carried out in accordance with the method described by Teterycz et al. [28]. A 15-member panel evaluated the pasta products. The panelists had been previously trained how to evaluate the sensory parameters of pasta: its appearance (the regularity of shape, lack of deformation, cracks, and scratches), color (should be regular and characteristic to raw materials used for pasta production), odor and taste (should be characteristic and similar to that of durum semolina pasta), hardness (evaluated as a resistance of cooked pasta to compression by the teeth), adhesiveness (evaluated by placing in the mouth, pressing it against the palate and determining the force required to remove it with the tongue), springiness (measured as the degree to which the product returns to its original shape after partial compression). The coded samples were cooked for the predetermined cooking time (CT) in a random order, placed in warm conditions until testing, and evaluated within a time no longer than 5 min after cooking. A five-point rating scale was used, in which 5 was the maximum score. Assuming that all the evaluated parameters had equal weight, the average sensory rating was calculated for each pasta sample. 

### 3.9. Statistical Analysis

The obtained results were subjected to statistical analysis using the statistical program STATISTICA 13.1 (StatSoft ©, Inc. Tulsa, OK, USA). All the experimental results were means (±S.D) from at least three assays. One-way analysis of variance (ANOVA) and Tukey’s posthoc test were chosen to compare the groups. The results were statistically different for *p*-values ≤ 0.05. Moreover, Pearson correlation coefficients (*p* < 0.05, *n* = 6) between all pasta features were determined. 

## 4. Conclusions

Stinging nettle is undoubtedly a raw material worth attention in the context of functional food design. The addition of nettle significantly (*p* ≤ 0.05) increased the content of ash and minerals, especially calcium, iron, potassium, and magnesium. Along with the increase in the content of nettle addition, the study revealed a statistically significant (*p* ≤ 0.05) increase in the total dietary fiber content, including the insoluble fiber fraction, as well as a significant (*p* ≤ 0.05) increase in the content of pigments—chlorophylls and carotenoids. The use of 1–5% nettle addition to pasta affects their cooking quality by extending the optimal cooking time and increasing the loss of dry matter. The lowest, statistically significant (*p* ≤ 0.05) values of starch hydrolysis and the predicted values of the glycemic index were found for pasta with a 3% addition of nettle. Although the consumer rating of nettle pasta was rated slightly lower than the semolina control pasta in terms of taste, the additive did not significantly deteriorate the overall sensory quality in both cooked and uncooked samples. Moreover, the addition of nettle at the level of 4% and 5% in cooked pasta had a positive effect on the acceptability of the color by consumers. In order to improve the stability of the antioxidant bioactive ingredients contained in the nettle, it is worth in future considering pasta production with the addition of nettle in the form of an extract.

## Figures and Tables

**Table 1:** The parameters of pasta extrusion.

Samples	Raw Materials (%)		Process Parameters		
	Semolina Durum	Stinging Nettle	Pressure(MPa)	Barrel Temperature (°C)	Extruder Output (kg h^−1^)
CON	100	-	12	27.9	28.26
N1	99	1	12.1	28.6	30.96
N2	98	2	12.5	28.3	30.48
N3	97	3	12.4	28.9	31.72
N4	96	4	12.5	28.5	30.84
N5	95	5	12.1	29.2	30.55

Explanation: CON—control sample; N—pasta with stinging nettle.

**Table 2:** Basic chemical composition of raw material and pasta samples.

Samples	Moisture	Protein	Fat	Ash	TDF	IDF	SDF	Digestible Carbohydrate
	**g 100 g^−1^ w.m.**				**g 100 g^−1^ d.m.**			
Raw materials								
Semolina durum	9.38 ^B^ ± 0.07	15.90 ^A^ ± 1.02	1.02 ^A^ ± 0.05	0.94 ^A^ ± 0.01	4.25 ^A^ ± 0.35	2.22 ^A^ ± 0.3	2.03 ^A^ ± 0.05	81.01
Stinging nettle	6.13 ^A^ ± 0.26	24.64 ^B^ ± 2.03	1.48 ^B^ ± 0.10	24.58 ^B^ ± 0.11	43.22 ^B^ ± 6.16	38.05 ^B^ ± 3.07	5.17 ^B^ ± 3.09	6.08
Pasta samples								
CON	10.11 ^c^ ± 0.2	16.21 ^a^ ± 0.19	0.19 ^a^ ± 0.01	1.00 ^a^ ± 0.02	5.10 ^a^ ± 0.77	2.29 ^a^ ± 1.02	2.81 ^a^ ± 0.26	78.40
N1	9.6 ^ab^ ± 0.03	16.12 ^a^ ± 0.17	0.17 ^a^ ± 0.01	1.25 ^b^ ± 0.02	5.46 ^a^ ± 0.01	2.26 ^a^ ± 0.12	3.20 ^a^ ± 0.10	77.00
N2	10.15 ^c^ ± 0.04	16.34 ^a^ ± 0.16	0.16 ^a^ ± 0.01	1.36 ^c^ ± 0.00	5.73 ^a^ ± 0.48	3.27 ^ab^ ± 0.02	2.45 ^a^ ± 0.46	76.41
N3	9.74 ^b^ ± 0.05	16.26 ^a^ ± 0.20	0.20 ^a^ ± 0.01	1.53 ^d^ ± 0.01	7.60 ^b^ ± 0.55	4.27 ^bc^ ± 0.36	3.33 ^a^ ± 0.19	74.41
N4	9.49 ^a^ ± 0.06	16.44 ^a^ ± 0.18	0.18 ^a^ ± 0.01	1.62 ^e^ ± 0.02	7.69 ^b^ ± 0.26	4.46 ^bc^ ± 0.18	3.23 ^a^ ± 0.43	74.07
N5	9.59 ^ab^ ± 0.26	16.66 ^a^ ± 0.14	0.14 ^a^ ± 0.01	2.04 ^f^ ± 0.00	8.82 ^c^ ± 0.10	5.63 ^c^ ± 0.36	3.19 ^a^ ± 0.47	72.34

Explanation: IDF—insoluble dietary fiber; SDF—soluble dietary fiber; TDF—total dietary fiber; CON—control sample; N—pasta with stinging nettle. Data are presented as mean (*n* = 3) ± standard deviation. Data values of each parameter with different superscript letters in the columns are significantly different (Tukey’s test, *p* ≤ 0.05).

**Table 3:** Concentration of the ions in the raw materials and uncooked pasta samples.

	Concentration of the Ions					
	Ca	Fe	K	Mg	Si	P
	mg·100 g^−1^ d.m.					g·kg^−1^ d.m.
Raw materials						
Semolina durum	33.33 ^A^ ± 2.36	3.99 ^A^ ± 0.28	26.04 ^A^ ± 1.84	46.35 ^A^ ± 3.28	16.00 ^A^ ± 1.13	2.32 ^A^ ± 0.16
Stinging nettle	7824.69 ^B^ ± 553.29	15.39 ^B^ ± 1.09	277.93 ^B^ ± 19.65	600.75 ^B^ ± 42.48	22.23 ^B^ ± 1.57	4.17 ^B^ ± 0.29
Pasta samples						
CON	30.50 ^a^ ± 0.30	2.38 ^a^ ± 0.10	270.50 ^a^ ± 1.92	51.54 ^a^ ± 0.16	2.30 ^a^ ± 0.24	2.39 ^a^ ± 0.08
N1	73.77 ^b^ ± 1.48	2.83 ^ab^ ± 0.04	292.36 ^ab^ ± 0.68	53.64 ^a^ ± 0.50	2.79 ^a^ ± 0.12	2.43 ^a^ ± 0.00
N2	88.47 ^c^ ± 4.97	2.89 ^ab^ ± 0.65	299.63 ^b^ ± 19.14	56.37 ^a^ ± 4.43	2.84 ^a^ ± 0.60	2.38 ^a^ ± 0.08
N3	131.99 ^d^ ± 7.49	2.98 ^ab^ ± 0.12	331.74 ^c^ ± 2.10	64.76 ^b^ ± 0.99	2.90 ^a^ ± 0.18	2.45 ^a^ ± 0.00
N4	120.31 ^d^ ± 3.98	2.99 ^ab^ ± 0.16	356.81 ^d^ ± 0.64	64.25 ^b^ ± 1.54	2.88 ^a^ ± 0.83	2.49 ^a^ ± 0.08
N5	175.89 ^e^ ± 6.85	3.23 ^b^ ± 0.25	372.91 ^d^ ± 2.82	72.80 ^c^ ± 1.20	2.98 ^a^ ± 0.09	2.49 ^a^ ± 0.08

Explanation: CON—control sample; N—pasta with stinging nettle; d.m.—dry matter. Data are presented as mean (*n* = 3) ± standard deviation. Data values of each parameter with different superscript letters in the columns are significantly different (Tukey’s test, *p* ≤ 0.05).

**Table 4:** Chlorophyll and carotenoid content.

Samples	Pigment Content		
	Chlorophyll *a*	Chlorophyll *b*	Carotenoids
	μg·g^−1^ d.m.		
Raw materials			
Semolina durum	22.42 ^b^ ± 0.28	3.42 ^a^ ± 0.79	1.99 ^a^ ± 1.01
Stinging nettle	2792.57 ^g^ ± 3.70	1997.67 ^f^ ± 7.46	146.24 ^g^ ± 2.60
Pasta samples			
CON	9.82 ^a^ ± 2.00	n.d.	1.05 ^a^ ± 0.01
N1	23.47 ^b^ ± 0.81	12.02 ^ab^ ± 0.09	2.52 ^ab^ ± 0.04
N2	48.26 ^c^ ± 0.51	22.58 ^bc^ ± 0.43	4.81 ^bc^ ± 0.37
N3	71.85 ^d^ ± 1.26	33.20 ^c^ ± 0.51	6.07 ^cd^ ± 0.27
N4	119.58 ^e^ ± 6.02	53.54 ^d^ ± 2.55	10.95 ^de^ ± 1.07
N5	160.74 ^f^ ± 0.51	76.84 ^e^ ± 2.82	13.35 ^f^ ± 1.39

Explanation: d.m.—dry matter; CON—control sample; N—pasta with stinging nettle. Data are presented as mean (*n* = 2) ± standard deviation. Data values of each parameter with different superscript letters in the columns are significantly different (Tukey’s test, *p* ≤ 0.05).

**Table 5:** Cooking quality of pasta samples.

Pasta Samples	Cooking Time (min)	Cooking Loss (% d.m.)	Cooking Weight Increase
CON	4.5 ^a^ ± 0.0	3.74 ^a^ ± 0.26	2.21 ^a^ ± 0.13
N1	5.0 ^ab^ ± 0.0	4.40 ^ab^ ± 0.35	2.32 ^a^ ± 0.01
N2	5.0 ^ab^ ± 0.5	4.33 ^ab^ ± 0.32	2.27 ^a^ ± 0.03
N3	5.5 ^bc^ ± 0.5	5.03 ^bc^ ± 0.49	2.45 ^a^ ± 0.30
N4	5.5 ^bc^ ± 0.0	5.83 ^bc^ ± 0.41	2.49 ^a^ ± 0.00
N5	6 ^c^ ± 0.0	6.19 ^c^ ± 0.08	2.45 ^a^ ± 0.05

Explanation: % d.m—% of dry matter; CON—control sample; N—pasta with stinging nettle. Data are presented as mean (*n* = 3) ± standard deviation. Data values of each parameter with different superscript letters in the columns are significantly different (Tukey’s test, *p* ≤ 0.05).

**Table 6:** Sensory quality of pasta samples.

Pasta Samples	Uncooked			Cooked						
	Appearance	Color	Odor	Appearance	Color	Taste	Odor	Hardness	Adhesiveness	Springiness
CON	4.93 ^b^ ± 0.26	4.93 ^c^ ± 0.26	5 ^b^ ± 0.00	4.93 ^a^ ± 0.26	4.53 ^ab^ ± 0.52	4.9 ^b^ ± 0.41	5 ^c^ ± 0.00	4.87 ^a^ ± 0.35	4.6 ^ab^ ± 0.63	4.73 ^ab^ ± 0.46
N1	4.33 ^a^ ± 0.62	4.2 ^a^ ± 0.41	4.73 ^b^ ± 0.46	4.86 ^a^ ± 0.52	4.27 ^a^ ± 0.59	4.07 ^a^ ± 0.96	4.2 ^ab^ ± 0.56	4.47 ^a^ ± 1.06	4.53 ^ab^ ± 0.64	4.47 ^a^ ± 0.52
N2	4.73 ^ab^ ± 0.46	4.4 ^ab^ ± 0.51	3.93 ^a^ ± 1.16	5.0 ^a^ ± 0.00	4.33 ^a^ ± 0.49	4.8 ^b^ ± 0.41	4.07 ^a^ ± 0.88	4.67 ^a^ ± 0.49	4.47 ^a^ ± 0.64	4.53 ^ab^ ± 0.52
N3	4.87 ^b^ ± 0.35	4.73 ^bc^ ± 0.46	4.4 ^ab^ ± 0.63	4.87 ^a^ ± 0.35	4.2 ^a^ ± 0.56	3.6 ^a^ ± 0.74	4.13 ^a^ ± 0.35	4.87 ^a^ ± 0.35	4.87 ^ab^ ± 0.35	4.93 ^b^ ± 0.26
N4	4.73 ^ab^ ± 0.46	4.53 ^abc^ ± 0.52	4.6 ^ab^ ± 0.51	4.87 ^a^ ± 0.35	4.6 ^ab^ ± 0.63	4.07 ^a^ ± 0.59	4.53 ^abc^ ± 0.52	4.87 ^a^ ± 0.35	4.87 ^ab^ ± 0.35	4.93 ^b^ ± 0.26
N5	4.8 ^ab^ ± 0.41	4.33 ^ab^ ± 0.49	4.73 ^b^ ± 0.59	4.8 ^a^ ± 0.41	4.93 ^b^ ± 0.26	3.87 ^a^ ± 0.52	4.73 ^bc^ ± 0.46	4.67 ^a^ ± 1.05	5 ^b^ ± 0.00	4.93 ^b^ ± 0.26

Explanation: CON—control sample; N—pasta with stinging nettle. Data are presented as mean (*n* = 15) ± standard deviation. Data values of each parameter with different superscript letters in the columns are significantly different (Tukey’s test, *p* ≤ 0.05).

## Data Availability

Not applicable.

## Sample Availability

Not available.
